# Cardiovascular Patterning as Determined by Hemodynamic Forces and Blood Vessel Genetics

**DOI:** 10.1371/journal.pone.0137175

**Published:** 2015-09-04

**Authors:** Gregory A. Anderson, Ryan S. Udan, Mary E. Dickinson, R. Mark Henkelman

**Affiliations:** 1 Department of Medical Biophysics, University of Toronto, Toronto, Ontario, Canada; 2 The Hospital For Sick Children, Toronto, Ontario, Canada; 3 Department of Molecular Physiology and Biophysics, Baylor College of Medicine, Houston, Texas, United States of America; Medical College of Wisconsin, UNITED STATES

## Abstract

**Background:**

Vascular patterning depends on coordinated timing of arteriovenous specification of endothelial cells and the concomitant hemodynamic forces supplied by the onset of cardiac function. Using a combination of 3D imaging by OPT and embryo registration techniques, we sought to identify structural differences between three different mouse models of cardiovascular perturbation.

**Results:**

*Endoglin* mutant mice shared a high degree of similarity to *Mlc2a* mutant mice, which have been shown to have a primary developmental heart defect causing secondary vessel remodeling failures. *Dll4* mutant mice, which have well-characterized arterial blood vessel specification defects, showed distinct differences in vascular patterning when compared to the disruptions seen in *Mlc2a* ^-/-^ and *Eng* ^-/-^ models. While *Mlc2a* ^-/-^ and *Eng* ^-/-^ embryos exhibited significantly larger atria than wild-type, *Dll4* ^-/-^ embryos had significantly smaller hearts than wild-type, but this quantitative volume decrease was not limited to the developing atrium. *Dll4* ^-/-^ embryos also had atretic dorsal aortae and smaller trunks, suggesting that the cardiac abnormalities were secondary to primary arterial blood vessel specification defects.

**Conclusions:**

The similarities in *Eng* ^-/-^ and *Mlc2a* ^-/-^ embryos suggest that *Eng* ^-/-^ mice may suffer from a primary heart developmental defect and secondary defects in vessel patterning, while defects in *Dll4* ^-/-^ embryos are consistent with primary defects in vessel patterning.

## Introduction

The cardiovascular system is the first organ system to arise in the mammalian embryo. In the mouse, the primitive heart becomes functional shortly after vasculogenesis has occurred on embryonic day (E) 8.0, and shortly thereafter circulation of the primitive erythroblasts begins [1,2]. It is now well recognized that the circulation of the blood is required for the normal patterning and remodeling of blood vessels [1–5]. Proper heart function is not only required for embryo survival, but mutations in several genes required solely for proper cardiac development and function, which are not expressed in blood vessels, also lead to defects in vascular remodeling, such as *Myosin light chain 2a* (*Mlc2a*) [6], *Nkx2*.*5* [7], *Alk3* [8], and titin [9]. Vascular remodeling defects in these mutants are likely caused by reduction of hemodynamic force, because experimentally reducing hematocrit [1], or aberrant blood cell development [10] can also lead to failed remodeling, while increasing viscosity in low hematocrit embryos can rescue this phenotype [1]. Normal vessel formation is dependent upon a complex network of signaling pathways [11,12]. Many genes required for proper vascular remodeling are expressed specifically in vascular endothelial cells and play a role in these cells directly, including Notch family members *Delta-like ligand 4* (*Dll4*) [13–15], *Jagged1* (*Jag1*) [16,17], the *Notch 1* and *Notch 4* receptors [18,19], as well as other receptors such as VEGFR2 (*Flk-1*) [20]. Mutations or inactivation of these genes lead to impaired vascular development. Interestingly, mutants in these genes also ultimately develop heart failure and lethality, since vessel remodeling is required to alleviate the resistance encountered as blood is pumped through such small capillaries. As such, it is not surprising that mutant mice with aberrant vessel patterning are so common, since normal development requires proper coordination between three systems: the heart, the blood, and the vasculature.

Advanced imaging tools have improved the ability to define cardiovascular abnormalities in mutant embryos [21–24]. In particular, optical projection tomography (OPT) [25] is a powerful imaging modality that is well suited to visualize and analyze mouse embryonic development. Our group has previously used OPT to obtain high resolution 3D images of the developmental stages of the mouse vasculature [26], to identify cardiovascular abnormalities in mouse mutant models [21,23], and to phenotype the neuroanatomy of the mouse brain [27]. Registration, a process that aims to properly align homologous points in anatomy between a source and a target image in 3D space, has also been utilized extensively by our group; we have developed novel techniques to register both the gross anatomy and the vascular trees of groups of mice. This allows for tracking and visualization of early cardiovascular development of the mouse embryo in its native 3D space, so that scaling and spatial orientation factors are no longer confounding issues [28]. By creating an averaged 3D model of vascular structures using genetically identical mouse embryos, we then can determine specific differences observed in mutant embryos using computation tools to compare and register 3D structures.

Here we sought to determine whether we could utilize our novel registration techniques to examine the growth of cardiovascular networks of three different mouse mutants. Myosin light chain 2a (*Mlc2a*) mutants have a heart specific functional defect (impaired atrial contraction) and show abnormal vessel remodeling resulting from reduced circulation [1,6]. Delta-like ligand 4 (*Dll4*) mutant embryos have been reported to show increased tip cell formation, enhanced angiogenesis, and the formation of chaotic, tortuous blood vessels, culminating in embryo death due to circulatory defects by E10.5 [13]. Finally, *Endoglin* (*Eng*) mutant mice are embryonic lethal by E10.5, but there is still some ambiguity about the precise reasons for lethality. Some groups report that *Eng* is necessary for proper mural cell investment in the growing vasculature during development [29], others believe *Eng* has a primary function in extraembryonic yolk sac vasculature development [30], while more recent studies have investigated the role of *Eng* in blood cell emergence [31]. By analyzing these three mouse mutants that succumb to embryonic lethality by different mechanisms, but at roughly the same developmental time point, we reasoned that this would allow us to differentiate, in a measurable and quantifiable manner, between mutations that primarily affect heart development (e.g. *Mlc2a*), and those that primarily affect blood vessel patterning (e.g. *Dll4*).

Utilizing the registration algorithms that we have developed, we were able to detect key structural differences between mutant and control mice for all three different genes. Interestingly, we identified a significantly larger heart volume, an atretic dorsal aorta, as well as an overall decrease in embryo volume in *Dll4* ^-/-^ mutant embryos that were not detected in *Mlc2a* ^-/-^ or *Eng* ^-/-^ mice. Overall, our analysis showed that *Eng* ^-/-^ mice share a high degree of similarity to *Mlc2a* ^-/-^ mice including a specific defect in heart structure detected in *Eng* ^-/-^ mutants that has not been previously identified. Although OPT is a terminal procedure, we show that by imaging multiple mice across a time series, it is possible to visualize and quantify small developmental differences between mouse models of cardiovascular disruption.

## Experimental Procedures

### Mouse lines

The *Eng* ^+/-^ mice were kindly provided by Dr. Michelle Letarte, and have been described previously [32]. They have been outbred to the C57Bl/6 background. The *Dll4* ^+/-^ mice were kindly provided by Dr. Janet Rossant, and have been described previously [13], though they have since been outbred to the FVB background which allows for total heterozygote viability. All somite-staged *Mlc2a* embryos were collected and kindly provided by Dr. Mary Dickinson, and have been described previously [6].

### Embryo collection and staining

All embryos were collected and stained as described previously[26]. Briefly, embryos were collected between embryonic day (E) 8.75–9.5 (13–28 somites). Noon of the day of vaginal plugging was considered to be E0.5. Embryos were dissected in PBS containing 0.1 M KCl (in order to stop the heart in diastole) and fixed in 4% paraformaldehyde for two hours. Endogenous peroxidase activity was quenched by immersing the embryos in 3% H_2_O_2_. Non-specific antibody staining was blocked by pre-incubating the embryos in 1% heat-inactivated fetal calf serum (FCS) and 1% normal goat serum. Embryos were then stained overnight with 5 μg/mL anti-PECAM-1 antibody (Mec13.3) (BD Pharmingen). The primary antibody was then detected by incubating the embryos overnight with an anti-rat horseradish peroxidase (HRP) secondary antibody (Biosource), followed by incubation with a tyramide-Cy3 reagent (1:50; Perkin-Elmer) for two hours at room temperature. The secondary antibody was then washed away with Tris-NaCl-Tween-20 (TNT) buffer overnight. All animal experiments in Texas were carried out in strict accordance with the recommendations in the Guide for the Care and Use of Laboratory Animals of the National Institutes of Health, and all animal research was conducted according to protocols approved by the Institutional Animal Care and Use Committee (IACUC) of Baylor College of Medicine. All animal experiments in Toronto were approved by the Animal Care Committee at Mount Sinai Hospital (Toronto, ON), and were conducted in accordance with guidelines developed by the Canadian Council on Animal Care.

### Optical projection tomography (OPT) of embryos

Optical projection tomography was performed as described previously [26] using a custom-built system [33]. Briefly, specimens were embedded in 1% low melting point agarose and subsequently cleared using a 1:2 mixture of benzyl alcohol and benzyl benzoate (BABB). The embryos were then suspended from a stepper motor and immersed in an optically flat cuvette containing BABB. An autofluorescence view was captured with a GFP excitation filter set in the illumination and detection light path, and a view of the PECAM-1 fluorescence from the specimen was captured using a Cy3 excitation filter set in the illumination and detection light path. The pixel size was equal to 7.4 μm and the acquisition time for a single filter set is approximately 20 minutes [33]. Reconstruction was performed using the standard convolution filtered back-projection algorithm [34]. The reconstruction of all slices produces a 3D volumetric representation of the embryo with isotropic pixel size of 4.45 μm.

### 3D registration of embryos

Autofluorescence scans of all embryos used for the registration were first subjected to a rigid body alignment (3 rotations, 3 translations) to orient the embryos into a preliminary atlas. This step removes postural and angular discrepancies. Next, all possible pair-wise 9 parameter affine transformations (3 translation, 3 rotations, and 3 scales) were computed, and a transform to an unbiased aggregate was created for each individual embryo. All scans were then averaged to create the first population average, which represents the average anatomy of the embryos after accounting for overall differences in body orientation and size. Next, an iterative 6-generation multi-scale, non-linear alignment procedure was computed, initially registering each embryo towards the 9 parameter registration atlas, and subsequently towards the atlas of the previous non-linear generation. Each step involves detailed matching of anatomical features using a coarse grid that becomes progressively finer with each non-linear step, finally ending at the resolution of the imaging voxels. All registrations were performed using the MNI autoreg tools [35]. The end result places all autofluorescence scans into precise alignment with each other in an unbiased manner. The deformation field for each individual embryo, which contains all information pertaining to the functions performed on it, was then applied to each embryo’s respective vasculature scan, thus approximately aligning all vasculature scans of the embryos with their autofluorescence scan counterparts. In order to account for any residual inconsistencies in alignment at this stage (since the vasculature has a very fine resolution), and to more precisely align the vascular trees, the vasculature scans were put through another iterative 6-generation multi-scale, non-linear alignment procedure as before (matching of vascular features using a coarse grid that becomes progressively finer with each non-linear step, finally ending at the resolution of the imaging voxels). Thus, all vascular scans are placed into precise alignment with each other in an unbiased fashion.

## Results

### Registration of mouse anatomy and vascular trees of *Dll4*, *Mlc2a*, and *Eng* embryos across four developmental time points

**Fig 1:**
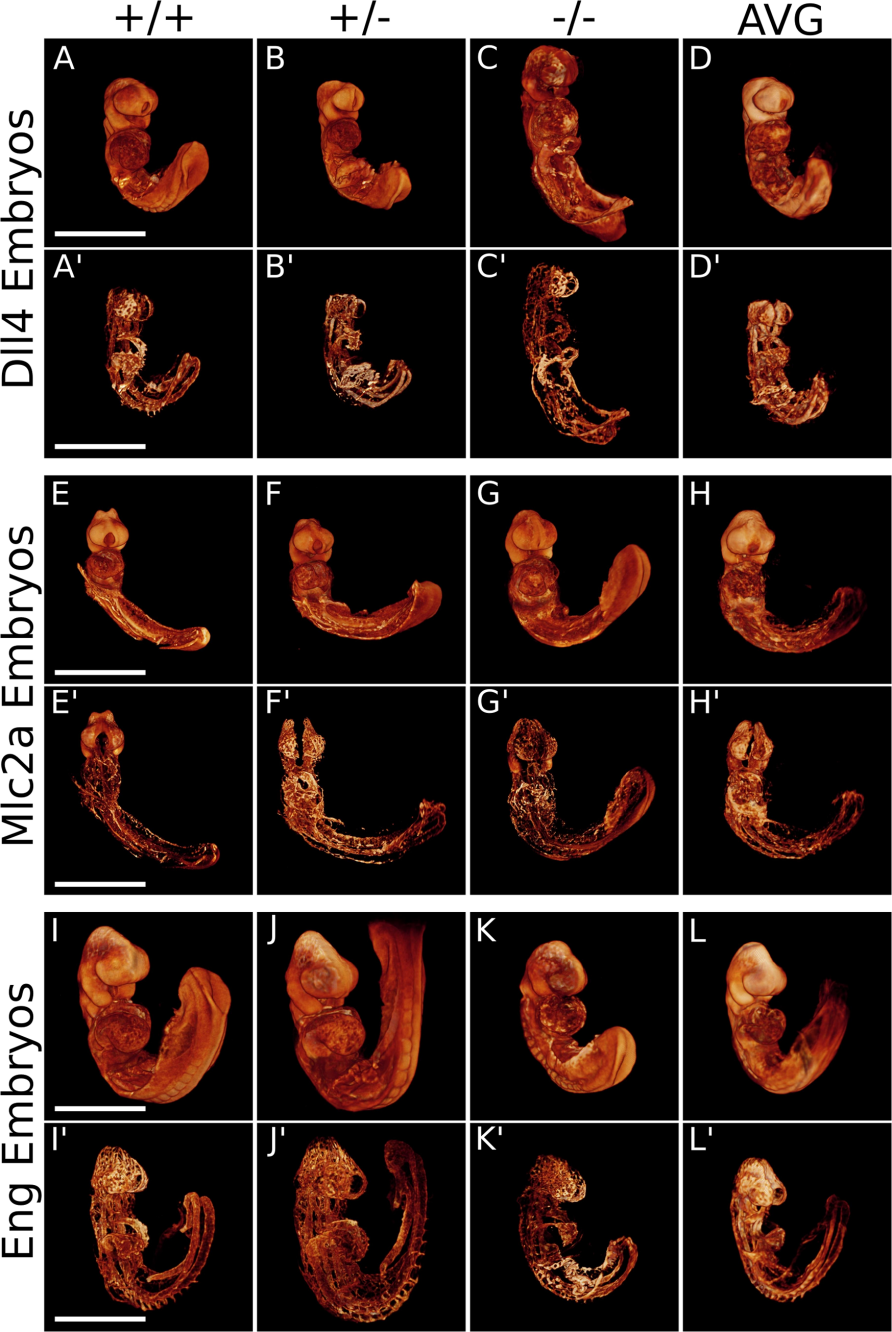
Registration of populations of *Dll4*, *Mlc2a*, and *Eng* embryos at 13–16 somites of age. Individual autofluorescence scans of representative embryos of 13–16 somite *Dll4* (A-C), *Mlc2a* (E-G), and *Eng* mice (I-K) are intrinsically aligned with their respective vasculature scans (A′-C′, E′-G′, and I′-K′). An average image of all the 13–16 somite embryos scanned was generated for each mouse line, for both the autofluorescence scans and the vascular scans respectively: *Dll4* (D and D′, n = 14), *Mlc2a* (H and H′, n = 10), and *Eng* (L and L′, n = 14). Scale bar = 500 μm.

*Dll4* ^-/-^, *Mlc2a* ^-/-^, and *Eng* ^-/-^ mice have all previously been shown to be embryonic lethal between E9.5—E10.5 [6,13,32]. We thus sought to analyze these three mouse lines from developmental time points that begin before the respective phenotypes are evident, to an endpoint when the embryos begin dying. We therefore collected embryos and separated them into four groups based on the number of somite pairs counted: 13–16 somites, 17–20 somites, 21–24 somites, and 25–28 somites. The embryos were stained with a fluorescent PECAM-1 antibody, which has been used extensively as a molecular marker of mature endothelial cells [26,36–39], in order to highlight the developing cardiovascular network. The embryos were then imaged with our custom-built OPT system [33] and then registered in three-dimensional space, as previously reported [28]. Previously, the earliest embryos we attempted to register were 20 somites in age [28]. Although challenging, due to their small size, autofluorescence scans and vascular scans (PECAM-1 staining) were amenable to registration as early as 13–16 somites across all genotypes (Fig 1). As shown in Fig 1, individual autofluorescence scans of representative embryos of 13–16 somite *Dll4* mice (A—C), *Mlc2a* mice (E—F), and *Eng* mice (I—K) are intrinsically aligned with their respective vasculature scans (A′—C′, E′—F′ and I′—K′). An average image of all the 13–16 somite embryos scanned was generated for each mouse line, for both the autofluorescence scans and the vascular scans: *Dll4* (Fig 1D and 1D′, n = 14), *Mlc2a* (Fig 1H and 1H′, n = 10), and *Eng* (Fig 1L and 1L′, n = 14).

The same procedure was applied to embryos in the other three developmental time point categories (S1–S3 Figs). Please see Table 1 for a summary of all embryos registered.

**Table 1:** Summary of all embryos in embryo registration groups.

	*Dll4* Mice				*Mlc2a* Mice				*Eng* Mice			
AGE GROUP	WT	HET	MUT	TOTAL	WT	HET	MUT	TOTAL	WT	HET	MUT	TOTAL
13–16 somites	6	5	3	14	3	2	5	10	5	6	3	14
17–20 somites	3	4	6	13	4	6	3	13	4	4	5	13
21–24 somites	4	8	8	20	3	8	5	16	2	5	6	13
25–28 somites	8	12	2	22	2	7	4	13	4	9	2	15

### Root mean squared displacement map alignment to a common average for each genotype

To define key differences in the structure of the heart and vascular system, we determined the root mean squared displacement (RMS) between cardiovascular maps generated from wild-type and heterozygous controls and those from mutant embryos. This approach provides a quantitative measure of differences in phenotype and the RMS displacement map can be visualized as a “heat map”, showing regions of high variability or where significant warping was required for registration. Thus, an area with a large voxel displacement represents an area of anatomical inconsistency among the individual embryos in the registration pipeline.

**Fig 2:**
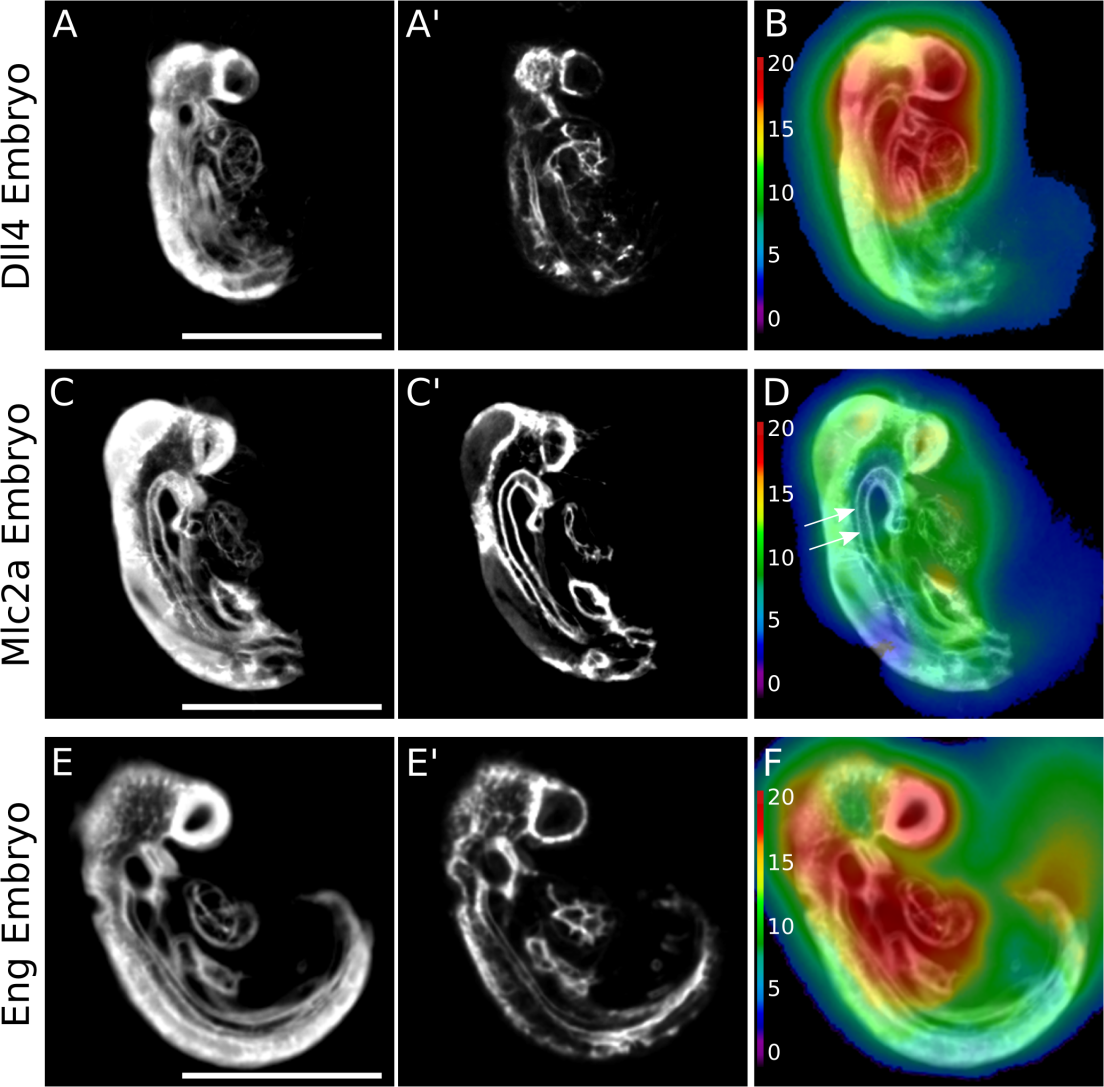
Root mean squared displacement maps of the 13–16 somite embryo registration pipelines. When a sagittal slice through the average autofluorescence image of the 13–16 somite *Dll4* population average (A) or the average vasculature image (A′) is overlaid with the root mean squared (RMS) displacement map generated in the registration pipeline, the movement of voxels required for proper alignment of the embryos can be visualized and quantified (B) (n = 14). The heat map scale bar (left side of B) indicates the voxel displacement in μm. The same is shown, respectively, for the 13–16 somite *Mlc2a* embryos (C, C′, and D), and the *Eng* embryos (E, E′, and F; n = 14). Scale bar = 500 μm.

Our 3D registration analysis identified the heart and the head as features with significant differences among *Dll4*, *Mlc2a*, and *Eng* embryos across all developmental time points. As an example, Fig 2 is an illustration of the pooled registration data of the 13–16 somite stage. Quantitative analysis revealed a high amount of voxel movement in both the head and heart region of the *Dll4* embryos (Fig 2B; n = 14), indicating that these two areas of the individual embryos were most variable. The analysis of *Mlc2a* embryos revealed the most movement of voxels within the heart of the average autofluorescence image (Fig 2D; n = 10). Note, however, that relatively little voxel movement is required to properly align the dorsal aorta in both the autofluorescence average image and the PECAM-1 image (Fig 2D, white arrows). *Mlc2a* embryos also appeared to have the most consistent phenotype, as they required the least voxel movement to align to a consensus average image compared with the *Dll4* (Fig 2B) or *Eng* embryos (Fig 2F). Analysis of *Eng* embryos revealed a similar pattern of voxel movement as the *Dll4* embryos at this time point: the most highly variable regions of the individual embryos, as illustrated by the average autofluorescence images (Fig 2F; n = 14), were the head and heart.

Embryos from each genotype were analyzed at all later developmental time points (S4–S6 Figs) and further structural divergence in the head and heart was observed between lines as the embryos become larger and more complex. The generalized voxel movement pattern in the RMS displacement maps of all embryos registered shows relatively large voxel displacement in the head and heart regions.

### Jacobian determinant maps quantitatively illustrate regions of mutant mice that differ from wild-type and heterozygote counterparts

**Fig 3:**
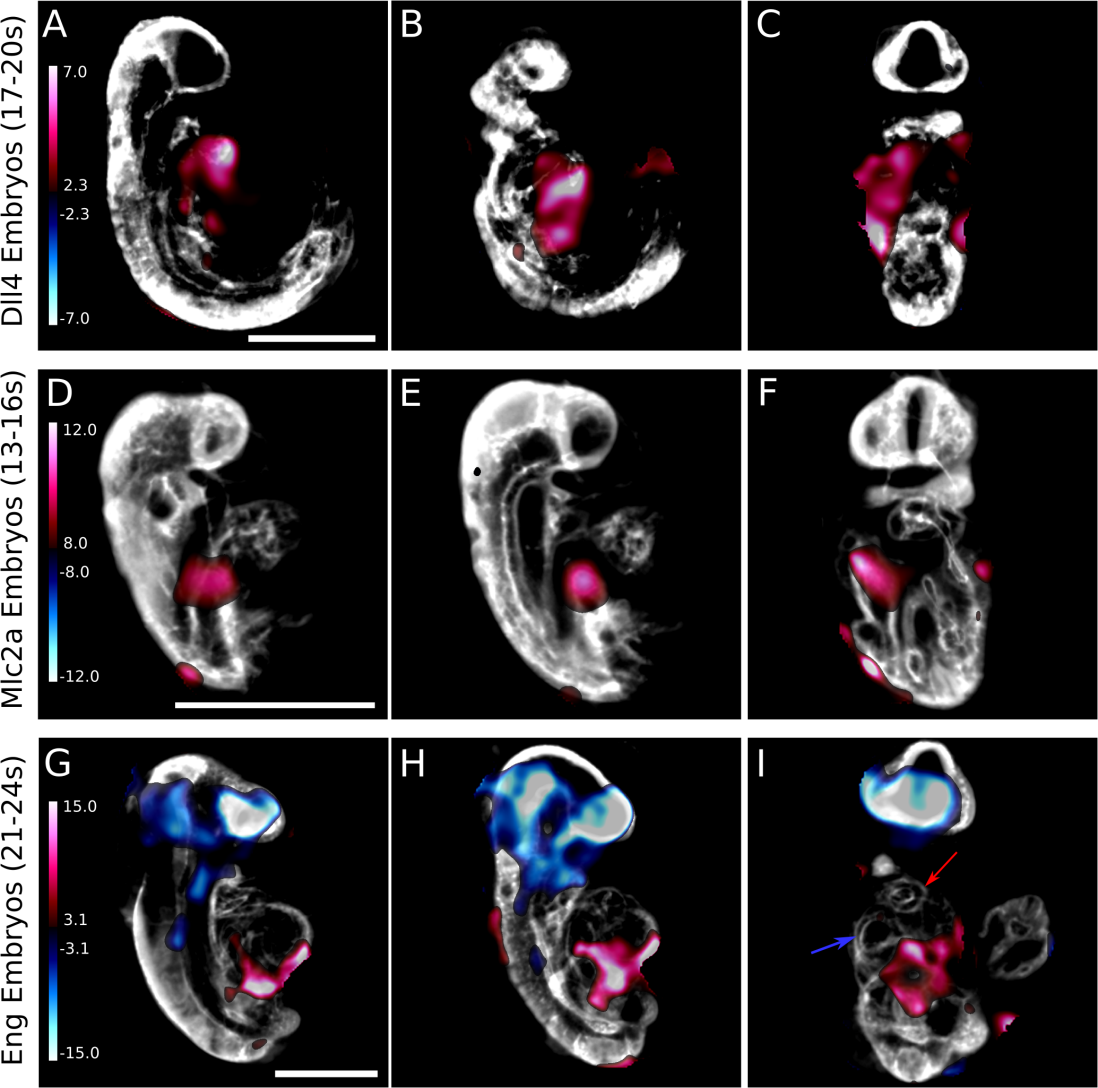
Jacobian determinant maps quantitatively illustrate mutant mouse differences. When the Jacobian determinant heat maps are overlaid over the population average autofluorescence image, statistically significant differences between mutant mice and their pooled wild-type and heterozygote counterparts are visible. For *Dll4* mice, statistically significant differences are visible at all time points, including the 17–20 somite stage (A-C). Two different sagittal sections (A, B) and a coronal section (C) through the autoflurorescence image reveal that *Dll4* mutants have generalized, whole-heart volume increases (red shading). *Mlc2a* mutant mice display significant volume increases in the atrium of the developing heart compared to their wild-type and heterozygote counterparts (red shading sagittal sections, D and E, and a coronal section, F) at the 13–16 somite stage. *Eng* mutant mice, similar to the *Mlc2a* mice, display volume increases in their developing atrium at the 21–24 somite stage (red shading in sagittal sections G and H), and significant volume decreases in their heads (blue shading in G-I). The developing ventricle (blue arrowhead in coronal section I) and developing outflow tract (red arrowhead in I) are unaltered. The colour bar presents the t-statistic of which the minimum corresponds to a false discovery rate (FDR) threshold of 10%. Scale bar = 500μm.

The RMS displacement map images are useful for highlighting the anatomical and vascular areas of difference between individuals within groups of mouse embryos that require a more intensive investigation. We were interested in discovering if, at a voxel level, we could pinpoint areas of the mouse anatomy that differed between wild-type and mutant mice in a statistically significant way. Thus, we first looked at the Jacobian determinant maps of each group of embryos in order to determine which, if any, anatomical regions differ in size between mutant and wild-type mice. The Jacobian determinant is a measure of the magnitude of deformation each voxel undergoes in order to reach the population average image during registration. Across all three mouse lines, in all four time points, there were no statistically significant differences between the wild-type and heterozygote mice (data not shown; from here on, the combined groups of wild-type and heterozygote mice will be designated as wild-type; see S1–S3 Tables for statistical data on numbers of mice dissected for *Dll4*, *Mlc2a*, and *Eng* mice respectively, and Mendelian ratios of the genotypes reported on). However, when the mutant mice were compared to the wild-type mice, some interesting observations were made. For *Mlc2a* mice, the only statistically significant differences occurred at the 13–16 somite stage. When the autofluorescence average images were overlaid with the Jacobian determinant map generated following registration, the mutant mice in this group showed significantly enlarged atria compared to the wild-type mice (Fig 3D–3F), as previously reported [6]. The red shading illustrates the areas where the mutants were enlarged, while blue shading illustrates the areas where the mutants were smaller compared to the wild-type. It is only in the early atrium that the mutants were enlarged. No statistically significant differences were observed at later time points.

The *Dll4* mice displayed a different phenotype. Statistically significant differences between mutant mice and their respective wild-type counterparts were visible at all developmental time points. Fig 3A–3C illustrates the differences found at the 17–20 somite stage. Generally, we identified increases in whole-heart mutant mouse volume in *Dll4* embryos that were not restricted to the atrium or ventricle specifically. Volume differences throughout the embryo between *Dll4* and wild-type embryos were further pronounced at both the 21–24 somite and 25–28 somite time points such that the entire autofluorescence average image is covered in extensive red and blue shading (data not shown).

Analysis of the *Eng* embryos showed similarity to the *Mlc2a* mice in the heart region. At the 21–24 somite time point, *Eng* ^-/-^ embryos have a significantly enlarged atrium compared to their wild-type counterparts (Fig 3G–3I). The volume increase is confined to the developing atrium (Fig 3G and 3H) while the ventricle (blue arrowhead) and outflow tract (red arrowhead) are not significantly altered. Furthermore, *Eng* ^-/-^ mice have a significantly smaller head (blue shading) than their wild-type counterparts, which was not observed in the *Mlc2a* mice. No statistically significant differences were noted at the other developmental time points.

### Signal intensity maps highlight regions of mutant mice that are mis-matched compared to wild-type

**Fig 4:**
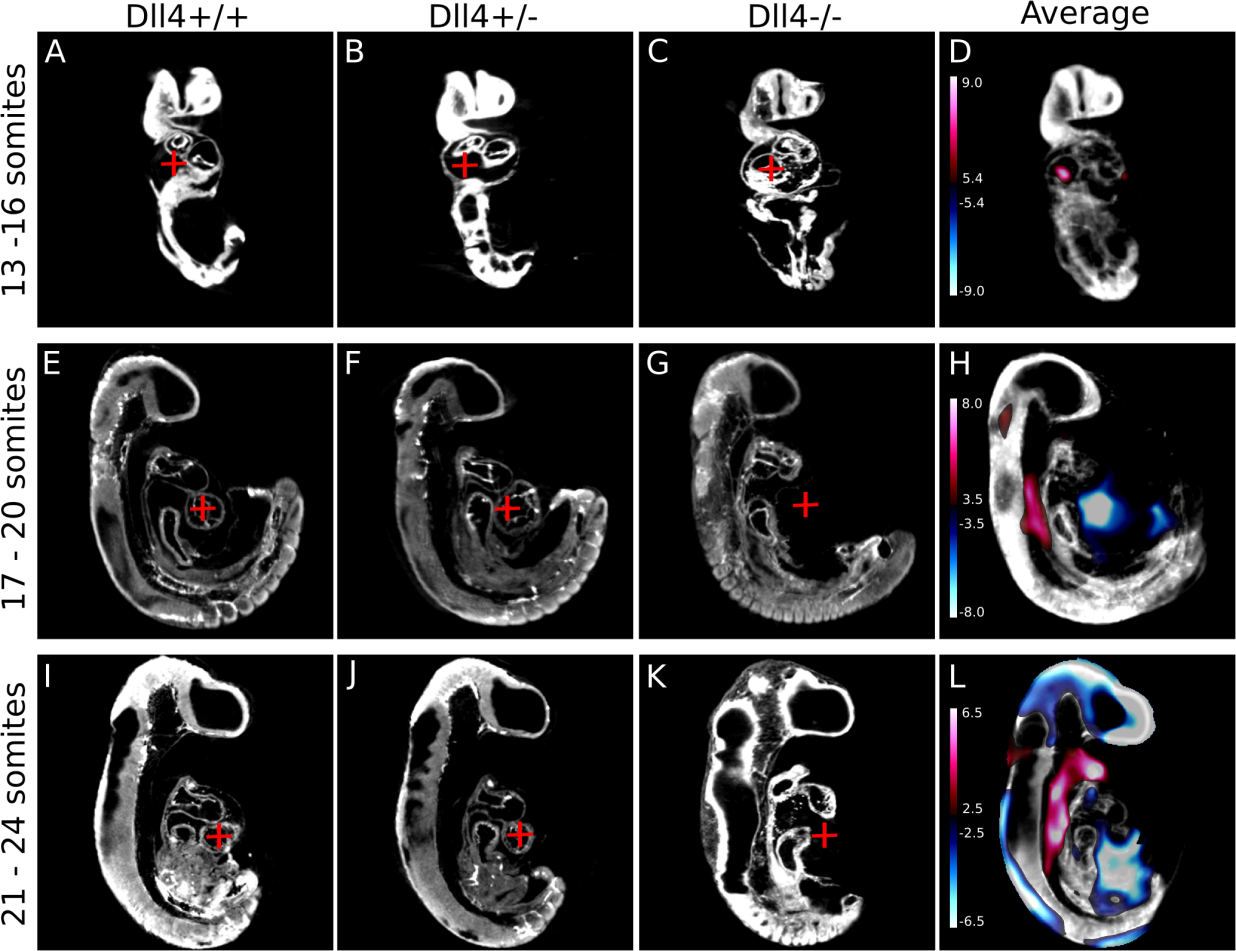
Signal intensity maps highlight regions of the *Dll4* mutant mice that are mis-matched compared to wild-type and heterozygote counterparts. (A–C) Individual wild-type, heterozygote, and mutant *Dll4* coronal sections through a 13–16 somite embryo, respectively, are shown while (D) is the average image from the registration pipeline. Because the heart of the *Dll4* ^-/-^ mouse (C) has not begun to undergo rightward looping, an area of signal increase (red cross-hairs in A-C, and red shading in D) is evident in the average image. This trend continues at the 17–20 somite stage, where sagittal sections through each genotype of the *Dll4* mice illustrate the lack of any heart structures in the mutants (red cross-hairs in E-G, and blue shading in H). The absence of the dorsal aorta is first noticeable at this time point as well; the red shading in H illustrates that signal exists in the *Dll4* ^-/-^ mouse, while an empty cavity is present in both the wild-type (E) and heterozygote (F) mouse. The persistence of a stunted, linear heart tube is even more pronounced at the 21–24 somite time point: the red cross-hairs in I-K illustrate the developing ventricle of the wild-type (I) and heterozygote (J) mouse, which is absent in the mutant mouse (K). The absence of the dorsal aorta is still noticeable as well (red shading in L). The colour bar presents the t-statistic of which the minimum corresponds to a FDR threshold of 10%. Scale bar = 500μm.

Jacobian determinant differences are useful at identifying small anatomical and vascular voxel differences within groups of registered mice. Some mutations, however, cause stunted or misshapen growth of developing organs [40], or can shift the developing anatomy into completely different locations. Thus, to further analyze structural differences between *Dll4* ^-/-^, *Mlc2a* ^-/-^, and *Eng* ^-/-^ embryos, we utilized signal intensity difference maps, which are essentially lack-of-correspondence maps, to highlight areas where there is an absence or presence (blue or red shading, respectively) of autofluorescence signal coming from the mutant mice compared to the wild-type mice. Using this method we detected that the *Dll4* ^-/-^ mice exhibited statistically significant signal intensity differences from their wild-type counterparts at every developmental time point tested. This was mainly due to the fact that the *Dll4* ^-/-^ hearts were stunted, linear, and did not undergo rightward looping from 13 somites onwards. This can be visualized quite nicely when individual embryos are placed side-by-side following registration. Fig 4A–4C are representative coronal sections of individual *Dll4* ^+/+^, *Dll4* ^+/-^, and *Dll4* ^-/-^ embryos respectively. Fig 4A and 4B show that the hearts of the *Dll4* ^+/+^ and *Dll4* ^+/-^ embryos had begun to undergo rightward looping, while the heart of the *Dll4* ^-/-^ mouse had not (notice that the red cross-hair in A and B is located underneath the early atrium, which had begun to loop, while the red cross-hair in C is located directly inside the atrium, because it had not moved yet). When the average autofluorescence image was overlaid with the signal intensity map (Fig 4D), a signal increase (red shading) was observed in the area where the red cross-hair is placed. This quantitatively illustrates that statistically significant positive signal exists in this area of the *Dll4* mutant mice that does not exist in the *Dll4* wild-type and heterozygote counterparts. Using this method, we did not, however, detect any statistically significant differences between the *Mlc2a* ^-/-^ mice and wild-type at any time point tested, nor were any differences observed between the *Eng* ^-/-^ mice and wild-type (data not shown).

Similar differences were present at later developmental time points. The blue-shaded area in Fig 4H indicates differences observed when the red cross-hair was placed in an area that corresponds to the rightward-looping heart’s ventricle in either the *Dll4* ^+/+^ and *Dll4* ^+/-^ embryos, but corresponds to empty space in the *Dll4* ^-/-^ embryo, because the mutant heart is still linear. Fig 4H also nicely shows the severely disrupted and atretic dorsal aorta in the *Dll4* ^-/-^ mice at this time point: the red shaded area indicates that the mutant mice showed a statistically significant signal increase where the dorsal aorta should exist. Fig 4G shows that the dorsal aorta was absent, and this cavity, which is present in Fig 4E and 4F, was filled in due to absence of this blood vessel. This had been previously demonstrated in a zebrafish [41] model of *Dll4* loss, and is suggested to be due to an increase in migration of arterial endothelial cells [16]. We also see a small area of statistically significant signal increase in the neck of the *Dll4* ^-/-^ mice, starting at the 17–20 somite stage (Fig 4H) and continuing on until the 21–14 somite stage (Fig 4L). This appears to be an area of edema that is present in the mutant mouse that is not present in the wild-type counterparts.

The same trend was identified as the *Dll4* mice reach the 21–24 somite stage (Fig 4I–4L). Once again, *Dll4* ^-/-^ embryos displayed a complete lack of signal (blue shading) where the rightward-looped heart ventricle should be, and an abundance of signal (red shading) where the dorsal aorta should exist. The *Dll4* ^-/-^ embryos were also found to be significantly growth retarded at this point, as illustrated by the signal decrease in the head of the average image (note the abundance of blue shading in the top of the head) and along the curvature of the tail. This indicated that the head and indeed the entire body was much smaller in the mutant *Dll4* embryos than wild-type. By the 25–28 somite developmental time point we identified even more severe differences between wild-type and mutant embryos using the same approach (data not shown).

### Side-by-side comparison of embryos highlight qualitative differences between mutant mice and their wild-type and heterozygote counterparts

**Fig 5:**
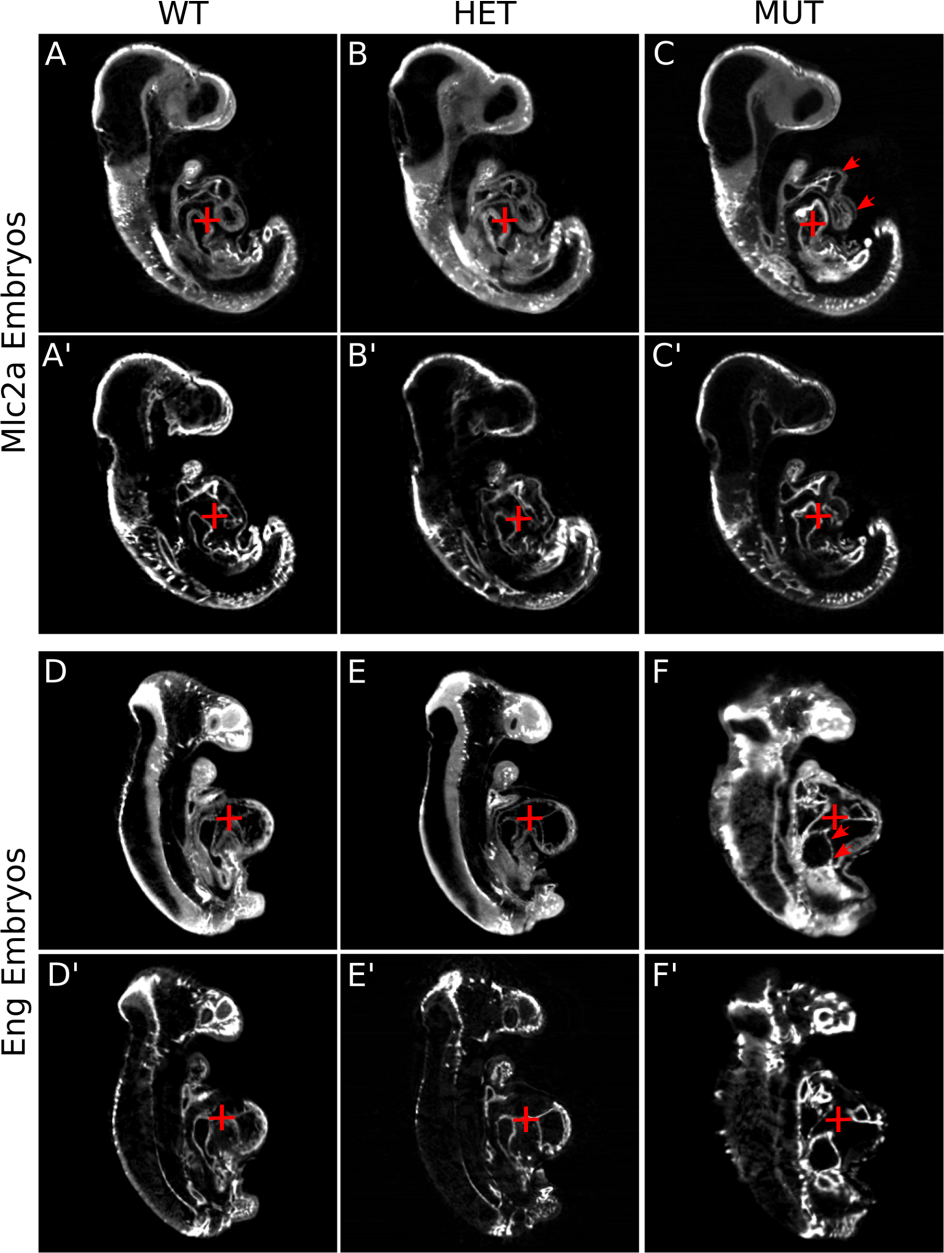
Side-by-side comparison of embryos highlight qualitative differences in mutant mice. Differences in heart size and structure of the *Mlc2a* and *Eng* mice becomes most evident at the 21–24 somite stage. (A-C) are sagittal slices through the autofluorescence images of an *Mlc2a* ^+/+^, *Mlc2a* ^+/-^, and *Mlc2a* ^-/-^ embryo, while (A′-C′) are the comparative slices through their respective vascular images. The red arrowheads highlight the fact that the ventricle and outflow tract have formed properly in all embryos, while the red cross-hair illustrates that the atrium is enlarged in the *Mlc2a* ^-/-^ mouse compared to the wild-type and heterozygote mouse. A more pronounced difference in heart structure is evident in the *Eng* mice at this time point. (D-F) are sagittal slices through the autofluorescence images of an *Eng* ^+/+^, *Eng* ^+/-^, and *Eng* ^-/-^ embryo, respectively, while (D′-F′) are the comparative slices through their respective vascular images. Not only is the atrium enlarged in *Eng* ^-/-^ mice (red arrowheads), but the mutant heart has maintained a more linear configuration (red cross-hair). Scale bar = 500μm.

As noted above, statistically significant Jacobian determinant differences between *Mlc2a* ^-/-^ and *Eng* ^-/-^ mice and their respective wild-type counterparts were only observed at one time point each. Similarly, no statistically significant signal intensity differences were noted for either mouse line at any time point. To determine if qualitative differences could be detected by side-by-side comparison that were not detected by automated methods, we re-analyzed *Mlc2a* and *Eng* embryos following image registration. For both the *Mlc2a* and *Eng* embryos, differences in heart size and structure were identified at the 21–24 somite developmental time point (Fig 5). The ventricle and outflow tract formed properly in all *Mlc2a* embryos (red arrowheads in Fig 5C), while the atrium was enlarged in the *Mlc2a* ^-/-^ mouse (red cross-hair in Fig 5A–5C) compared to the wild-type embryos.

A similar, but more pronounced difference in heart structure was evident in the *Eng* mice at the 21–24 somite time point (Fig 5D–5F). In the case of the *Eng* mice, not only was the atrium enlarged in the *Eng* ^-/-^ mice (red arrowheads in Fig 5F), but the mutant heart had maintained a more linear configuration than its wild-type counterpart (Fig 5F, note how much lower the atrium was in the *Eng* ^-/-^ embryo, and how different is the angle at which the atrioventricular canal connects the atrium to the ventricle (red cross-hair)).

### Registration of mutant mice across all three mouse lines reveals that *Dll4* mutants differ significantly from *Mlc2a* and *Eng* mice

**Fig 6:**
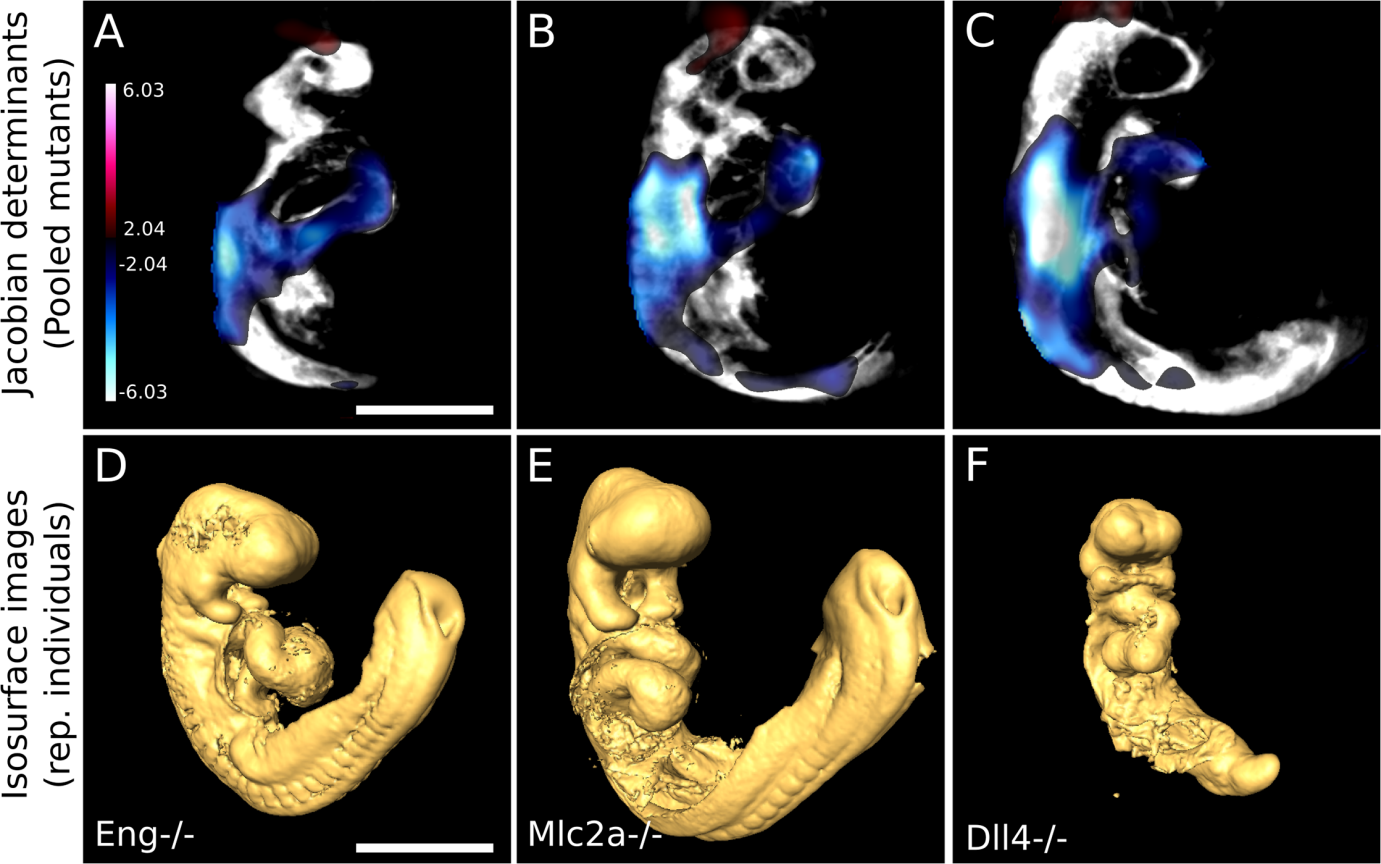
Registration of mutant mice across all three mouse lines reveals that *Dll4* have significantly different Jacobian determinant maps compared to *Mlc2a* and *Eng* mice. *Dll4* ^-/-^ mice were compared to the pooled population of *Mlc2a* ^-/-^ and *Eng* ^-/-^ mice at all time points after registration. (A-C) are sagittal slices through the average 17–20 somite autofluorescence image of all the mutants in the pipeline overlaid with the Jacobian determinant heat map generated during the registration, and in each slice it is evident that the *Dll4* mice are smaller than the other mutants in both the trunk and heart regions (blue shading). This is also qualitatively obvious when isosurface images of representative *Eng* ^-/-^ (D), *Mlc2a* ^-/-^ (E), and *Dll4* ^-/-^ mice are shown side-by-side. The *Dll4* ^-/-^ mouse also has a significantly growth-delayed, linear heart tube (compare heart tube geometry between D-F). The colour bar presents the t-statistic of which the minimum corresponds to a FDR threshold of 10%. Scale bar = 500μm.

Our previous analyses identified differences between each mutant mouse and its corresponding wild-type counterparts. Since it was identified that the *Dll4* embryos displayed characteristics different to that of the *Mlc2a* and *Eng* embryos, we next sought to quantify differences between the mutants alone. We next registered just the mutant embryos from the three different genotypes to each other, at each of the four developmental time points. Interestingly, statistically significant differences were only noticeable when the *Dll4* embryos were compared to the *Mlc2a* and *Eng* mutant mice pooled together. At the 13–16 somite time point, no significant differences were observed between the *Dll4* mice and the *Mlc2a* and *Eng* mice, when either the Jacobian determinants or signal intensity maps were evaluated. However, when the *Dll4* mutant mice were compared to the pooled *Mlc2a* and *Eng* mice at the 17–20 somite stage, significant differences were noted in the resulting Jacobian determinants of the registration pipeline. In Fig 6A–6C it is evident that the *Dll4* ^-/-^ mice were significantly smaller than their counterpart *Mlc2a* ^-/-^ and *Eng* ^-/-^ mice in both the trunk and heart regions at this time point (blue shading). This is qualitatively obvious when isosurface images of representative *Eng* ^-/-^ (Fig 6D), *Mlc2a* ^-/-^ (Fig 6E), and *Dll4* ^-/-^ (Fig 6F) are shown side-by-side. The *Dll4* ^-/-^ mice were more growth retarded than either the *Mlc2a* ^-/-^ or the *Eng* ^-/-^, and displayed a delayed, linear heart tube (compare the heart tube looping geometry of Fig 6F to 6D or 6E).

**Fig 7:**
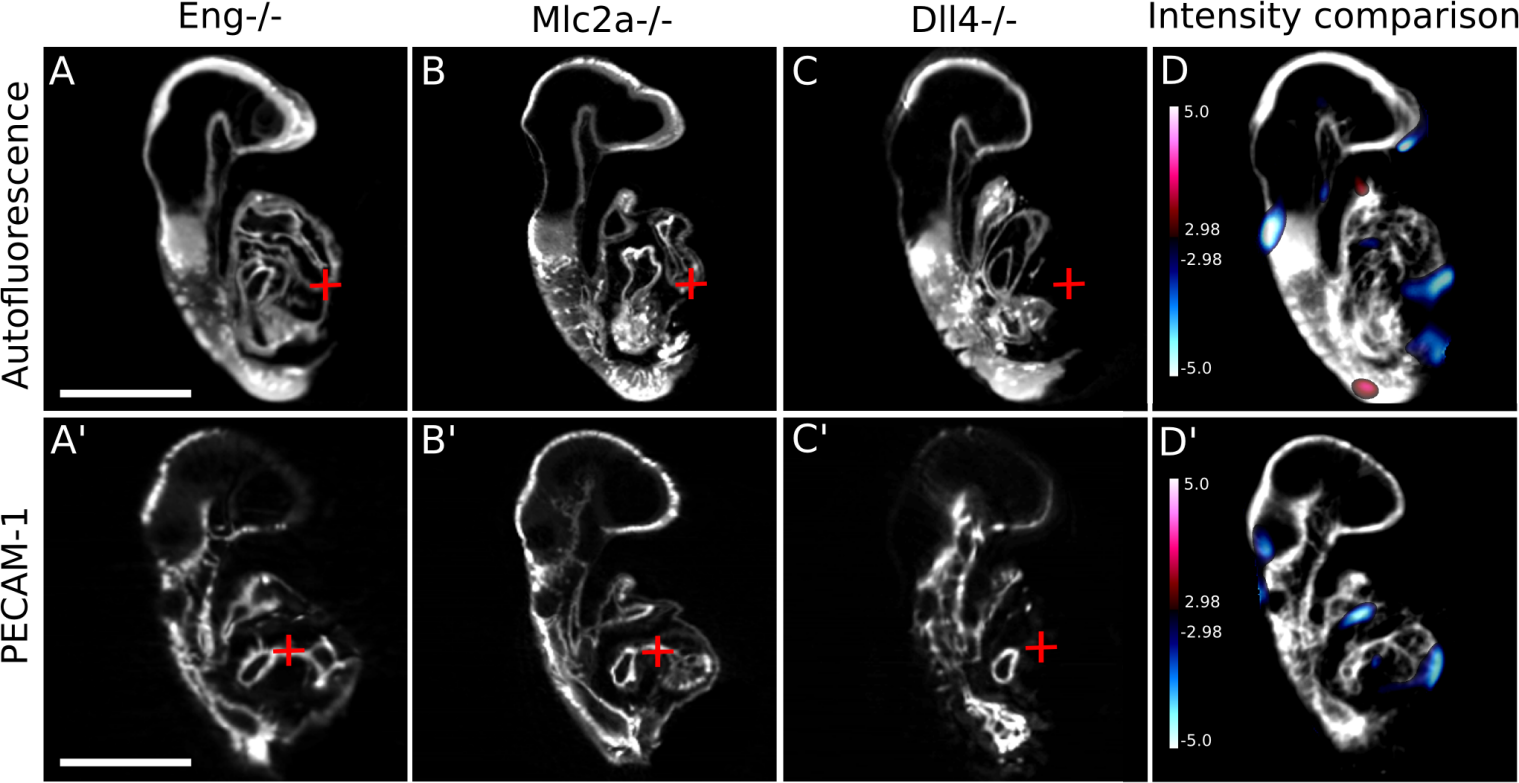
Registration of mutant mice across all three mouse lines reveals that *Dll4* have significantly different signal intensity maps compared to *Mlc2a* and *Eng* mice. *Dll4* ^-/-^ mice were compared to the pooled population of *Mlc2a* ^-/-^ and *Eng* ^-/-^ mice at all time points after registration. (A-C) are sagittal slices through an individual *Eng*-/-, *Mlc2a* ^-/-^, and *Dll4* ^-/-^ mouse, respectively. The red cross-hair corresponds to the common ventricle in both the *Eng* ^-/-^ (A) and *Mlc2a* ^-/-^ (B) mouse, but not in the *Dll4* ^-/-^ mouse (C) because its heart tube is still linear. (D) illustrates this statistically: the average autofluorescence map has been overlaid with the signal intensity map, and the *Dll4* ^-/-^ mice display a lack of signal intensity (blue shading) in the area where the common ventricle should be forming. Slices through the vascular scan of each embryo confirms this finding (A′—D′). The red cross-hair is placed in the centre of the antrioventricular canal of the *Eng* ^-/-^ mouse (A′), the *Mlc2a* ^-/-^ mouse (B′), but not in the *Dll4* ^-/-^ mouse (C′). Statistically, this is illustrated when the average vascular scan is overlaid with the signal intensity map (D′): the *Dll4* ^-/-^ mice display a lack of signal (blue shading) in the outer ventricle area of the heart compared to the pooled *Eng* ^-/-^ and *Mlc2a* ^-/-^ embryos. The colour bar presents the t-statistic of which the minimum corresponds to a FDR threshold of 10%. Scale bar = 500μm.

The heart tube looping defect of the *Dll4* ^-/-^ mice was even more pronounced at the 21–24 somite time point. Fig 7 illustrates the significant signal intensity differences between the *Dll4* ^-/-^ mice and the pooled *Mlc2a* ^-/-^ and *Eng* ^-/-^ mice. The red cross-hair corresponds to the common ventricle in both the *Eng* ^-/-^ and *Mlc2a* ^-/-^ mouse, yet not in the *Dll4* ^-/-^ mouse, due to the fact that the heart tube is still linear. This is shown statistically in Fig 7D, in which the average autofluorescence image has been overlaid with the signal intensity map: the *Dll4* ^-/-^ mice display a lack of signal intensity (blue shading) in the area where the common ventricle should be forming. Slices through the vascular data of each embryo, in the atrioventricular canal region of the same embryos, confirm this finding. The red cross-hair was placed squarely in the centre of the atrioventricular canal of the *Eng* ^-/-^ mouse (Fig 7A′) and the *Mlc2a*-/- mouse (Fig 7B′), but not in the *Dll4* ^-/-^ mouse (Fig 7C′), because the heart tube is still linear. Again, this is shown statistically in Fig 7D′, in which the average vascular scan of the mutants has been overlaid with the signal intensity map: the *Dll4* ^-/-^ display a lack of signal (blue shading) in the outer ventricle area of the heart compared to the pooled *Mlc2a* ^-/-^ and *Eng* ^-/-^ embryos.

## Discussion

Modern developmental biology and biomedical research rely on a profound understanding of the underlying genetic and molecular pathways that govern cellular differentiation, tissue remodeling, and morphogenesis. Traditional 2D developmental biology techniques are often limited in their ability to analyze the morphology and anatomy of mutant mouse embryos, because of their inability to preserve the innate 3D structure of the source tissue. Even 3D confocal imaging, which can provide high resolution images of the underlying vasculature of mouse embryos suffers from light penetration issues in older embryos. OPT has shown tremendous potential in analyzing embryo development, mouse brain development, and malformation syndromes [21,26–28], because it is ideally suited to visualize and analyze the complex three-dimensional structure of small samples with high resolution. Presented here is an in-depth analysis of three different mouse mutant models of cardiovascular malformation. We showed that embryo registration is possible in embryos as young as 13 somites, and in populations of mice that include homozygous mutants. Our embryo registration algorithm provided us with a powerful tool to analyze the anatomy and cardiovascular maps of each embryo, and make quantitative and qualitative comparisons between the wild-type, heterozygous, and mutant mice in any given group.

The time points we chose for analysis encompass the entire breadth of progression of each mutation, from when the individual mutations may begin to become mildly visible (13–16 somites), up to the point where the mutation is severe, and mutant mice begin to die (25–28 somites). Following registration, the RMS displacement maps were analyzed in order to determine which voxels had to move the most in order to align properly in the population average. When these RMS displacement maps were superimposed upon the population average image generated, the areas of the individual embryos that were most quantifiably variable became obvious, providing context for further analyses, pointing to areas where the mutation was having its biggest effect. We showed here that the *Dll4* mice required the most voxel displacement in the head, branchial arches, dorsal aorta, and developing heart in order to reach a consensus population average at all time points. The *Eng* mice required relatively large voxel displacement in the head, branchial arches, and developing heart, while the *Mlc2a* embryos required the most voxel displacement in the head and heart, but required very little voxel displacement in the branchial arches or dorsal aorta in order to reach a consensus population average at all time points. This suggested that this highly-patterned and prominent blood vessel is properly formed even in the *Mlc2a* mutant mice of this heart-specific mutant mouse model.

Following registration there are many quantifiable metrics from the registration pipeline that can be obtained and visualized in order to accurately assess the progression of the specific mutation in question. Jacobian determinant maps, and signal intensity maps are two such metrics. We showed that the *Mlc2a* ^-/-^ mice, at 13–16 somites, are significantly larger in the developing atrium than their wild-type counterparts. This would be expected, given that the *Mlc2a* ^-/-^ mice have been shown [1,6] to have enlarged, non-functional atria by other groups. Although *Mlc2a* ^-/-^ mice have also been shown to have underdeveloped trabeculae in their developing ventricles [42], we were not able to delineate that phenotype here in any statistical manner. This is due to the fact that trabeculation is not a consistently patterned process, and thus the vasculature of the trabeculae are not amenable to registration processes. A similar story was observed in the *Eng* ^-/-^ mice, albeit at a later time point: *Eng* ^-/-^ mice have significantly larger atria than their wild-type counterparts at 21–24 somites, while the rest of the heart remained unaffected. Unlike the *Mlc2a* mutants, the *Eng* ^-/-^ mice also suffered from a significantly smaller head, likely due to growth retardation, as well as a failure of the perineural vascular plexus to form properly at this developmental time point [43]. The *Dll4* ^-/-^ mice, however, displayed significant differences from their wild-type counterparts at all time points tested. Specifically, at the 17–20 somite stage, *Dll4* ^-/-^ mice had significantly smaller hearts than the wild-type mice. This volume decrease was not limited to the developing atrium, but was instead spread generally over the entire volume of the heart. This trend continued at all other time points, and also included volume decreases of the mutants in the trunk of the embryos as growth retardation became evident. Thus, the *Eng* ^-/-^ mice appeared to share more in common with the *Mlc2a* ^-/-^ than the *Dll4* ^-/-^ mice when analyzed quantitatively.

Signal intensity difference maps are also incredibly useful, in that they can be used to quantitatively analyze misregistration outcomes. In our study we identified statistically significant signal intensity differences at every time point for the *Dll4* mice, but not for the *Eng* or *Mlc2a* mice. Coronal sections for individual *Dll4* ^+/+^, *Dll4* ^+/-^, and *Dll4* ^-/-^ embryos, for instance, clearly showed that the *Dll4* ^-/-^ mouse heart had retained a more linear, unlooped heart configuration. Because the mutant heart was not undergoing rightward looping, as it should have been at this stage of development, the signal intensity map located this area on the population average as hyper-intense (red shading) in a statistically significant way. This trend continued at later time points for the *Dll4* mutants; the hearts of the wild-type embryos continued to develop and loop, while the *Dll4* ^-/-^ heart remained stunted and linear. Wild-type embryos clearly showed the heart bulging out from the body, while the *Dll4* ^-/-^ embryo did not display this. The signal intensity maps highlighted these areas as regions of statistically significant hypo-intensity (blue shading). Aside from the heart, another effect of the *Dll4* mutation became evident, namely the loss of a lumenized dorsal aorta. The signal intensity maps clearly indicated an area of hyper-intensity on the population average images that corresponded to the developing dorsal aorta. Since the dorsal aorta had failed to form and lumenize properly, in the *Dll4* ^-/-^ image this showed up as regular tissue instead of an empty cavity, thus becoming highlighted as a hyper-intensity on the population average. This phenotype had been discovered in zebrafish models of *Dll4* loss previously [41], and now we have shown here a mouse model of this phenotype. No such statistically significant signal intensity differences were observed in the *Mlc2a* or *Eng* mouse lines.

By directly comparing the three mouse mutants, we also showed that developmental differences existed between the *Mlc2a* ^-/-^ and *Eng* ^-/-^ mice and their respective wild-type counterparts. The *Mlc2a* ^-/-^ embryo was shown to have an enlarged atrium, while the rest of the heart and body plan appeared similar in morphological structure to the *Mlc2a* ^+/+^ and *Mlc2a* ^+/-^ embryos. A similar, but more exaggerated story was seen in the *Eng* ^-/-^ mice: the atrium of the *Eng* ^-/-^ mouse was enlarged, but the overall structure of the heart was also more linear and diffuse. It has previously been reported that *Eng* ^-/-^ mice suffer from dilation of their outflow tracts and enlarged ventricles [44], yet we did not find either of these structures to be significantly altered when analyzed in a group-wise manner. That we did not observe these abnormalities in this study suggests that these mutant phenotypes may be variably penetrant at the developmental time points analyzed in this study, which would preclude them from being flagged in a statistically significant manner using our technique. Thus, the *Eng* ^-/-^ embryos suffered a similar, but more extreme, fate as the *Mlc2a* ^-/-^ embryos.

We show here a method in which to make more quantifiable conclusions about the morphological differences in mouse mutant models. *Eng* ^-/-^ mice were shown to share more statistical commonalities with *Mlc2a* ^-/-^ mice. *Dll4* ^-/-^ mice presented different phenotypic abnormalities than the *Mlc2a* ^-/-^ and *Eng* ^-/-^ mice. We believe that the heart formation defect seen in the *Dll4* ^*-/-*^ mice was a downstream, secondary defect as a result of the primary arterial specification defect. It has previously been shown that mutations that have a primary effect on vasculature formation routinely lead to downstream heart formation defects [3]. Similarly, ligation studies have also shown that decreases in heart flow and ejection fraction volumes lead to downstream heart defects [45]. That *Eng* ^-/-^ mice appeared to suffer from a primary heart developmental defect is in line with our previous results obtained both in *in vitro* and *in vivo* [46]. There we showed that *Eng* ^-/-^ embryonic stem cells could contribute to the growing vasculature of chimeric mice but not to the developing endocardial cushions of the heart, and that *Eng* ^-/-^ embryos had significantly lower expression of *Snai1* and significantly higher expression of *Vegf* in their heart tissue compared to both wild-type and heterozygote littermates. However, it must be noted that our technique is not able to take into account any defects that arise due to yolk sac vascular defects. Because the yolk sac vasculature does not arise in a highly-directed or patterned manner, these vascular structures not amenable to registration techniques. It has been previously reported that *Eng* ^-/-^ mice suffer from yolk sac vascular defects at early stages of development [47], and since we did not, and cannot analyze these structures in a statistical manner, we cannot preclude their involvement the phenotypes that we have observed here.

Embryo registration techniques are powerful tools for developmental biologists, as they allow for intuitive, statistical comparisons between groups of similarly-staged embryos, and provide results in the native, three-dimensional space of the given embryos. It is a robust method that can aid developmental biologists in answering long-standing questions about phenotypes of interest with statistical rigour, and is applicable to many imaging modalities. Later staged embryos, imaged by micro-CT, for instance, are amenable to this analysis [40,48]. It would also be very interesting to apply this registration analysis technique to data acquired by high-resolution episcopic microscopy (HREM) [49], since HREM data is near-histological in terms of resolution and applicable to all developmental stages of relevant model organisms. We presented here the analysis of three models of mouse cardiovascular defects using our method, and conclude that because *Eng* ^-/-^ mice share a statistically similar phenotype to that of *Mlc2a* ^-/-^ mice, that it is possible that they suffer from a primary heart developmental defect. Conversely, the heart defects seen in the *Dll4* ^-/-^ mice, which presented in a quantifiably different manner, were secondary to a primary arterial specification defect.

## Supporting Information

S1 Fig Registration of populations of *Dll4*, *Mlc2a*, and *Eng* embryos at 17–20 somites of age. Individual autofluorescence scans of representative embryos of 17–20 somite *Dll4* (A-C), *Mlc2a* (E-G), and *Eng* mice (I-K) are intrinsically aligned with their respective vasculature scans (A′-C′, E′-G′, and I′-K′). An average image of all the 17–20 somite embryos scanned was generated for each mouse line, for both the autofluorescence scans and the vascular scans respectively: *Dll4* (D and D′, n = 13), *Mlc2a* (H and H′, n = 13), and *Eng* (L and L′, n = 13). Scale bar = 500 μm.
(TIF)

S2 Fig Registration of populations of *Dll4*, *Mlc2a*, and *Eng* embryos at 21–24 somites of age. Individual autofluorescence scans of representative embryos of 21–24 somite *Dll4* (A-C), *Mlc2a* (E-G), and *Eng* mice (I-K) are intrinsically aligned with their respective vasculature scans (A′-C′, E′-G′, and I′-K′). An average image of all the 21–24 somite embryos scanned was generated for each mouse line, for both the autofluorescence scans and the vascular scans respectively: *Dll4* (D and D′, n = 20), *Mlc2a* (H and H′, n = 16), and *Eng* (L and L′, n = 13). Scale bar = 500 μm.
(TIF)

S3 Fig Registration of populations of *Dll4*, *Mlc2a*, and *Eng* embryos at 25–28 somites of age. Individual autofluorescence scans of representative embryos of 25–28 somite *Dll4* (A-C), *Mlc2a* (E-G), and *Eng* mice (I-K) are intrinsically aligned with their respective vasculature scans (A′-C′, E′-G′, and I′-K′). An average image of all the 25–28 somite embryos scanned was generated for each mouse line, for both the autofluorescence scans and the vascular scans respectively: *Dll4* (D and D′, n = 22), *Mlc2a* (H and H′, n = 13), and *Eng* (L and L′, n = 15). Scale bar = 500 μm.
(TIF)

S4 Fig Root mean squared displacement maps of the 17–20 somite embryo registration pipelines. When a sagittal slice through the average autofluorescence image of the 17–20 somite *Dll4* population average (A) or the average vasculature image (A′) is overlaid with the RMS displacement map generated in the registration pipeline, the movement of voxels required for proper alignment of the embryos can be visualized and quantified (B). The heat map scale bar (left side of B) indicates the voxel displacement in μm. The same is shown, respectively, for the 17–20 somite *Mlc2a* embryos (C, C′, and D), and the *Eng* embryos (E, E′, and F). Scale bar = 500 μm.
(TIF)

S5 Fig Root mean squared displacement maps of the 21–24 somite embryo registration pipelines. When a sagittal slice through the average autofluorescence image of the 21–24 somite *Dll4* population average (A) or the average vasculature image (A′) is overlaid with the RMS displacement map generated in the registration pipeline, the movement of voxels required for proper alignment of the embryos can be visualized and quantified (B). The heat map scale bar (left side of B) indicates the voxel displacement in μm. The same is shown, respectively, for the 21–24 somite *Mlc2a* embryos (C, C′, and D), and the *Eng* embryos (E, E′, and F). Scale bar = 500 μm.
(TIF)

S6 Fig Root mean squared displacement maps of the 25–28 somite embryo registration pipelines. When a sagittal slice through the average autofluorescence image of the 25–28 somite *Dll4* population average (A) or the average vasculature image (A′) is overlaid with the RMS displacement map generated in the registration pipeline, the movement of voxels required for proper alignment of the embryos can be visualized and quantified (B). The heat map scale bar (left side of B) indicates the voxel displacement in μm. The same is shown, respectively, for the 25–28 somite *Mlc2a* embryos (C, C′, and D), and the *Eng* embryos (E, E′, and F). Scale bar = 500 μm.
(TIF)

S1 Table Summary of all *Dll4* embryos dissected. A table containing the information of all *Dll4* embryos dissected during the course of this study.
(PDF)

S2 Table Summary of all *Mlc2a* embryos dissected. A table containing the information of all *Mlc2a* embryos dissected during the course of this study.
(PDF)

S3 Table Summary of all *Eng* embryos dissected. A table containing the information of all *Eng* embryos dissected during the course of this study.
(PDF)
