# Supplementary figures and images for: Cardiovascular Patterning as Determined by Hemodynamic Forces and Blood Vessel Genetics
Source: PLoS One. 2015 Sep 4;10(9):e0137175. doi: 10.1371/journal.pone.0137175 (PMC4560395; doi:10.1371/journal.pone.0137175)

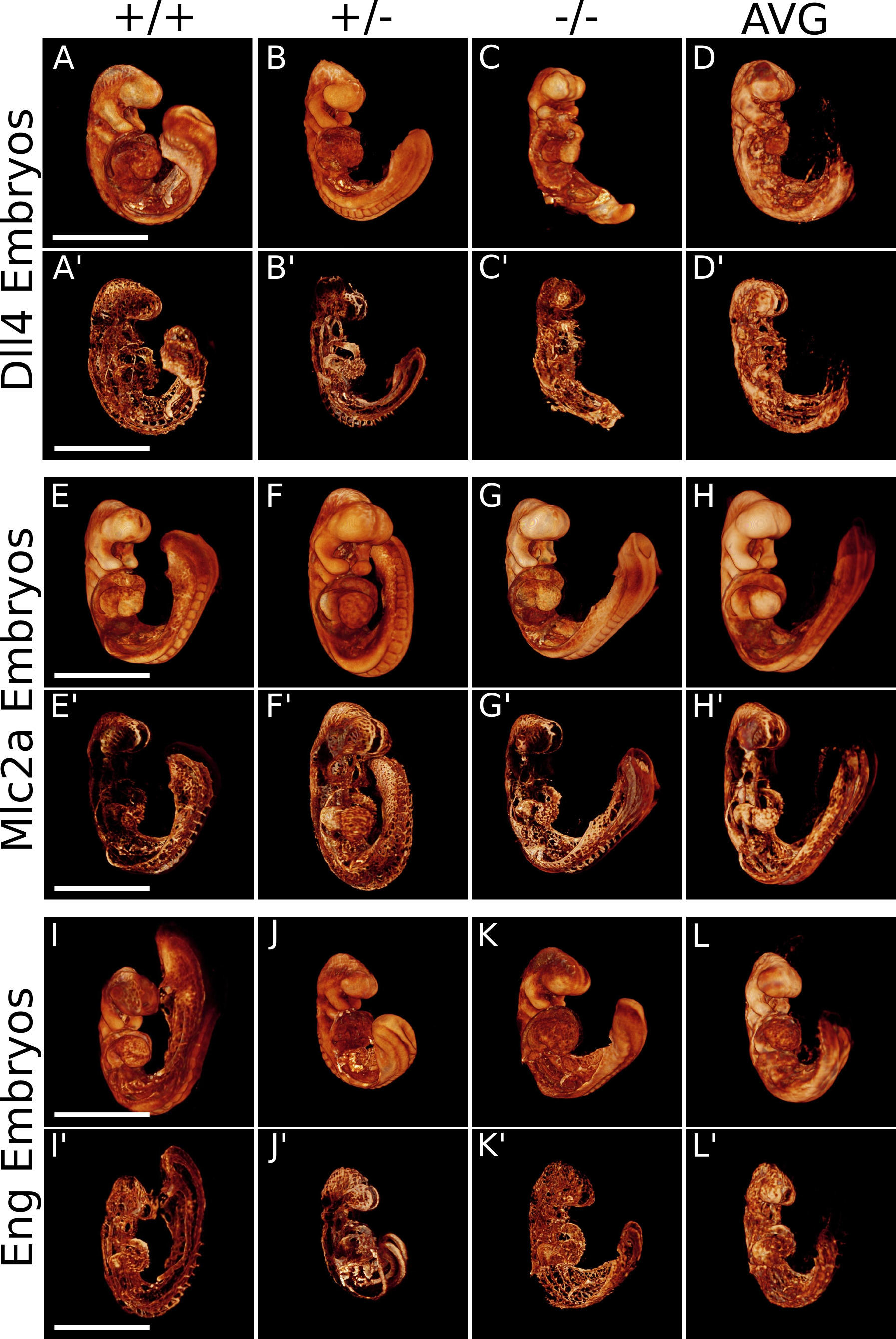

Supplement: S1 Fig — Individual autofluorescence scans of representative embryos of 17–20 somite Dll4 (A-C), Mlc2a (E-G), and Eng mice (I-K) are intrinsically aligned with their respective vasculature scans (A′-C′, E′-G′, and I′-K′). An average image of all the 17–20 somite embryos scanned was generated for each mouse line, for both the autofluorescence scans and the vascular scans respectively: Dll4 (D and D′, n = 13), Mlc2a (H and H′, n = 13), and Eng (L and L′, n = 13). Scale bar = 500 μm. (TIF) [file pone.0137175.s001.tif]

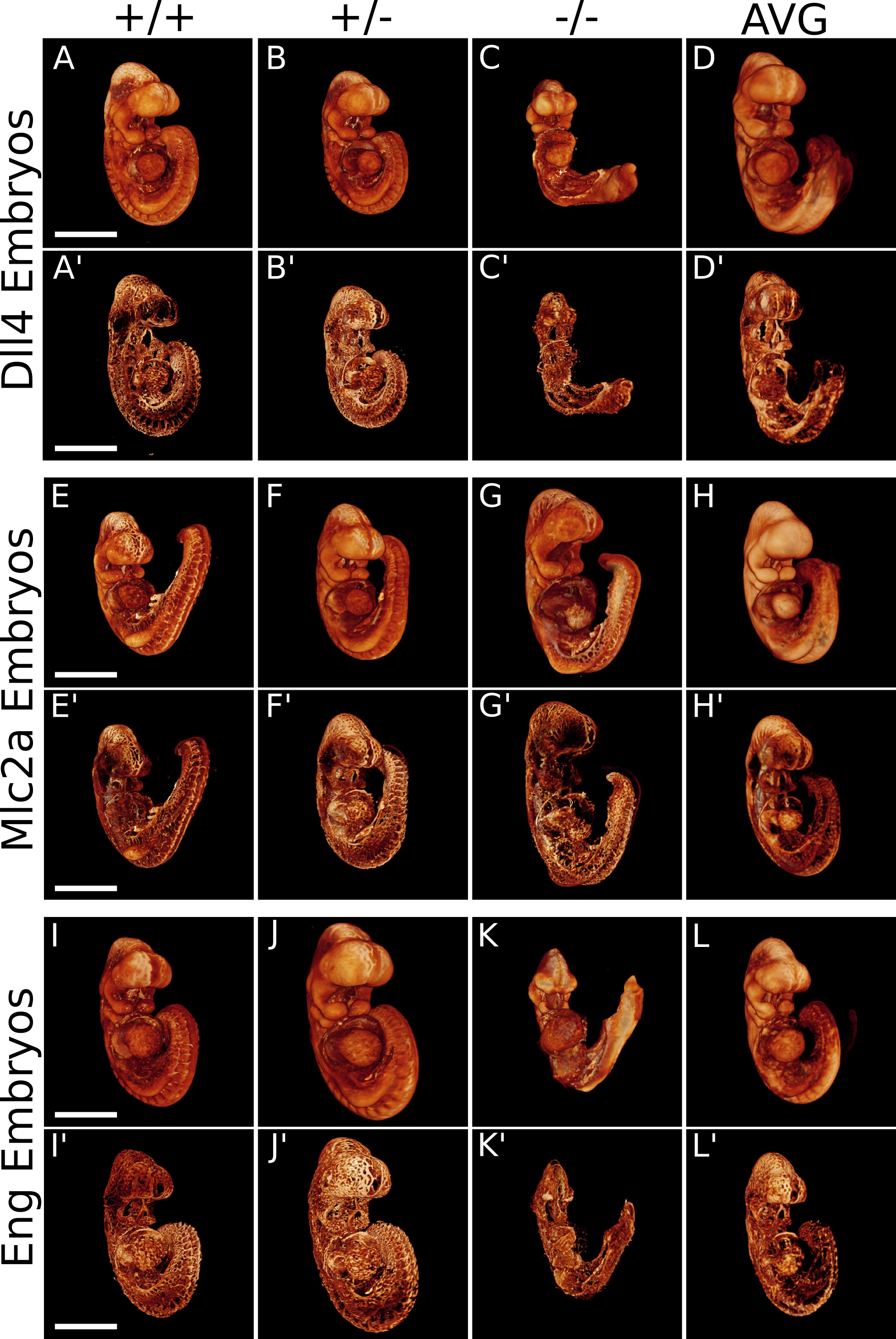

Supplement: S2 Fig — Individual autofluorescence scans of representative embryos of 21–24 somite Dll4 (A-C), Mlc2a (E-G), and Eng mice (I-K) are intrinsically aligned with their respective vasculature scans (A′-C′, E′-G′, and I′-K′). An average image of all the 21–24 somite embryos scanned was generated for each mouse line, for both the autofluorescence scans and the vascular scans respectively: Dll4 (D and D′, n = 20), Mlc2a (H and H′, n = 16), and Eng (L and L′, n = 13). Scale bar = 500 μm. (TIF) [file pone.0137175.s002.tif]

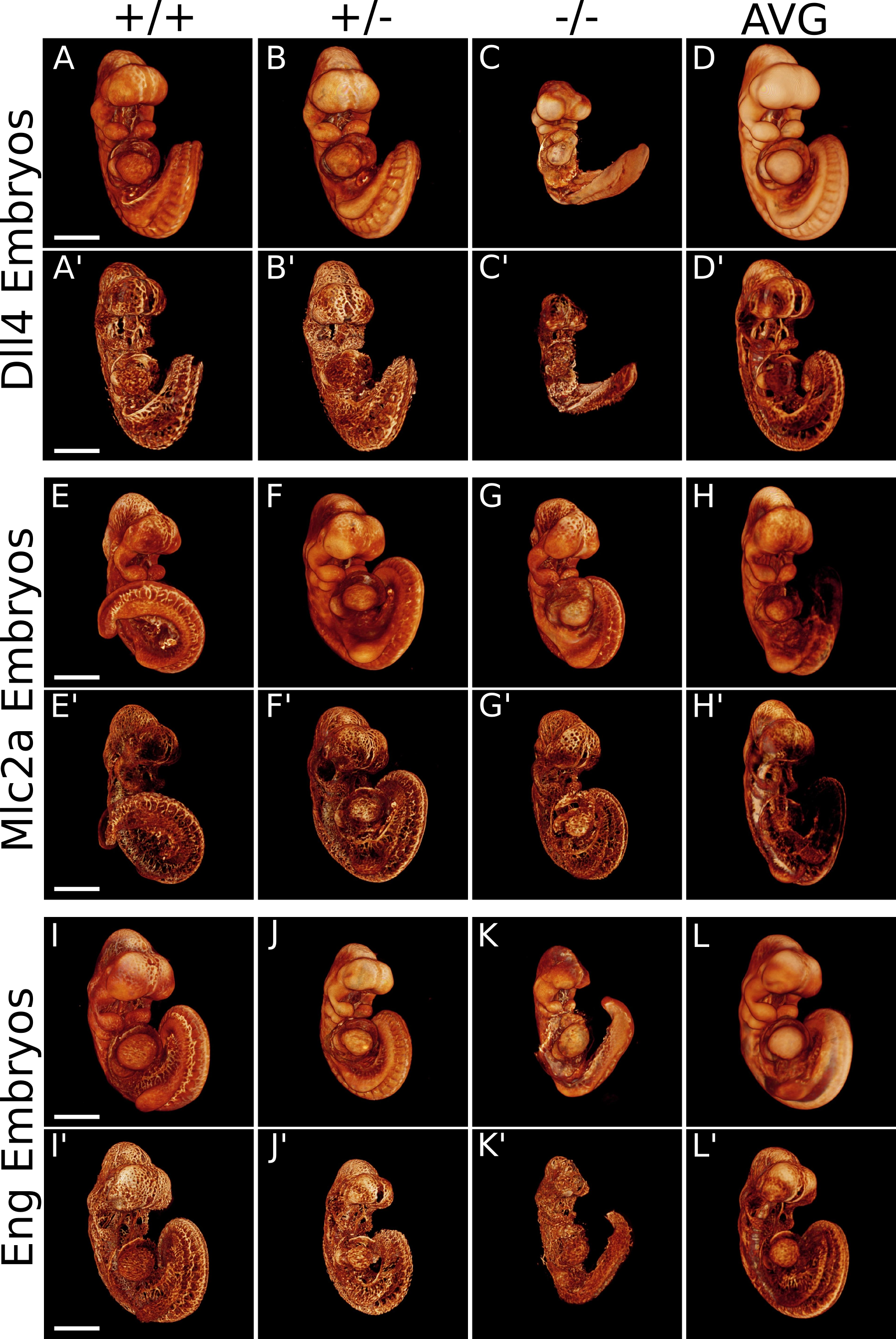

Supplement: S3 Fig — Individual autofluorescence scans of representative embryos of 25–28 somite Dll4 (A-C), Mlc2a (E-G), and Eng mice (I-K) are intrinsically aligned with their respective vasculature scans (A′-C′, E′-G′, and I′-K′). An average image of all the 25–28 somite embryos scanned was generated for each mouse line, for both the autofluorescence scans and the vascular scans respectively: Dll4 (D and D′, n = 22), Mlc2a (H and H′, n = 13), and Eng (L and L′, n = 15). Scale bar = 500 μm. (TIF) [file pone.0137175.s003.tif]

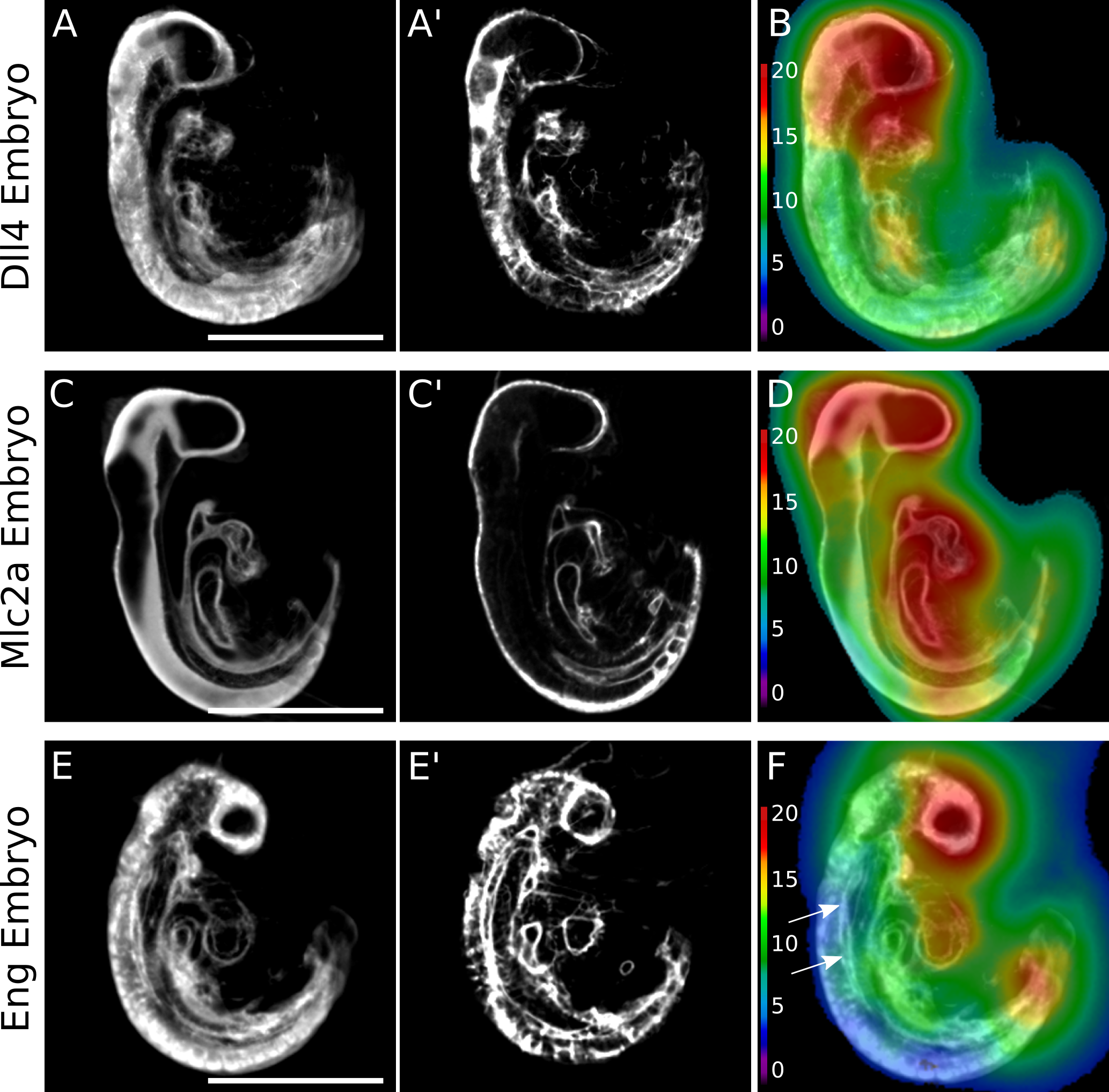

Supplement: S4 Fig — When a sagittal slice through the average autofluorescence image of the 17–20 somite Dll4 population average (A) or the average vasculature image (A′) is overlaid with the RMS displacement map generated in the registration pipeline, the movement of voxels required for proper alignment of the embryos can be visualized and quantified (B). The heat map scale bar (left side of B) indicates the voxel displacement in μm. The same is shown, respectively, for the 17–20 somite Mlc2a embryos (C, C′, and D), and the Eng embryos (E, E′, and F). Scale bar = 500 μm. (TIF) [file pone.0137175.s004.tif]

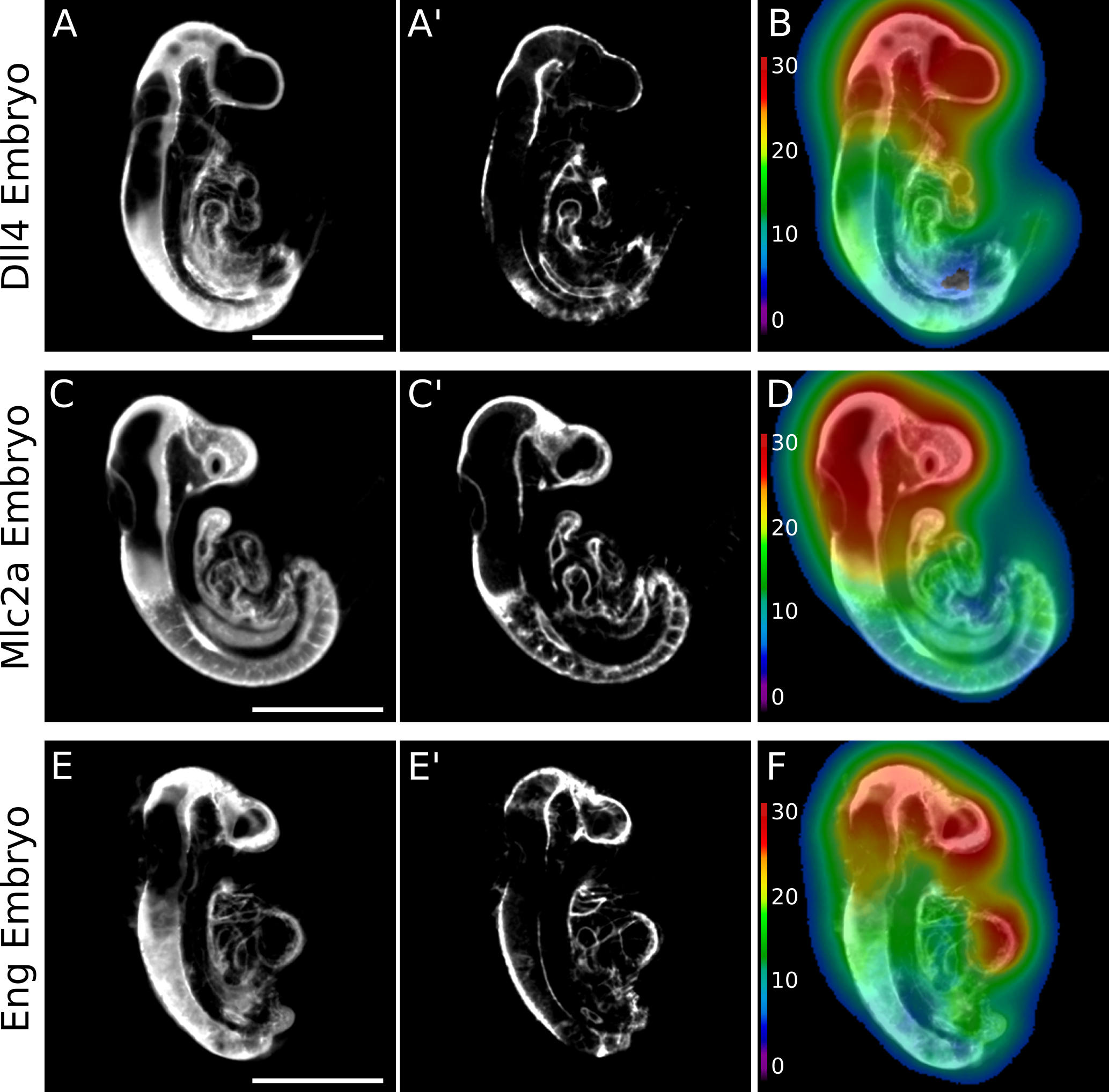

Supplement: S5 Fig — When a sagittal slice through the average autofluorescence image of the 21–24 somite Dll4 population average (A) or the average vasculature image (A′) is overlaid with the RMS displacement map generated in the registration pipeline, the movement of voxels required for proper alignment of the embryos can be visualized and quantified (B). The heat map scale bar (left side of B) indicates the voxel displacement in μm. The same is shown, respectively, for the 21–24 somite Mlc2a embryos (C, C′, and D), and the Eng embryos (E, E′, and F). Scale bar = 500 μm. (TIF) [file pone.0137175.s005.tif]

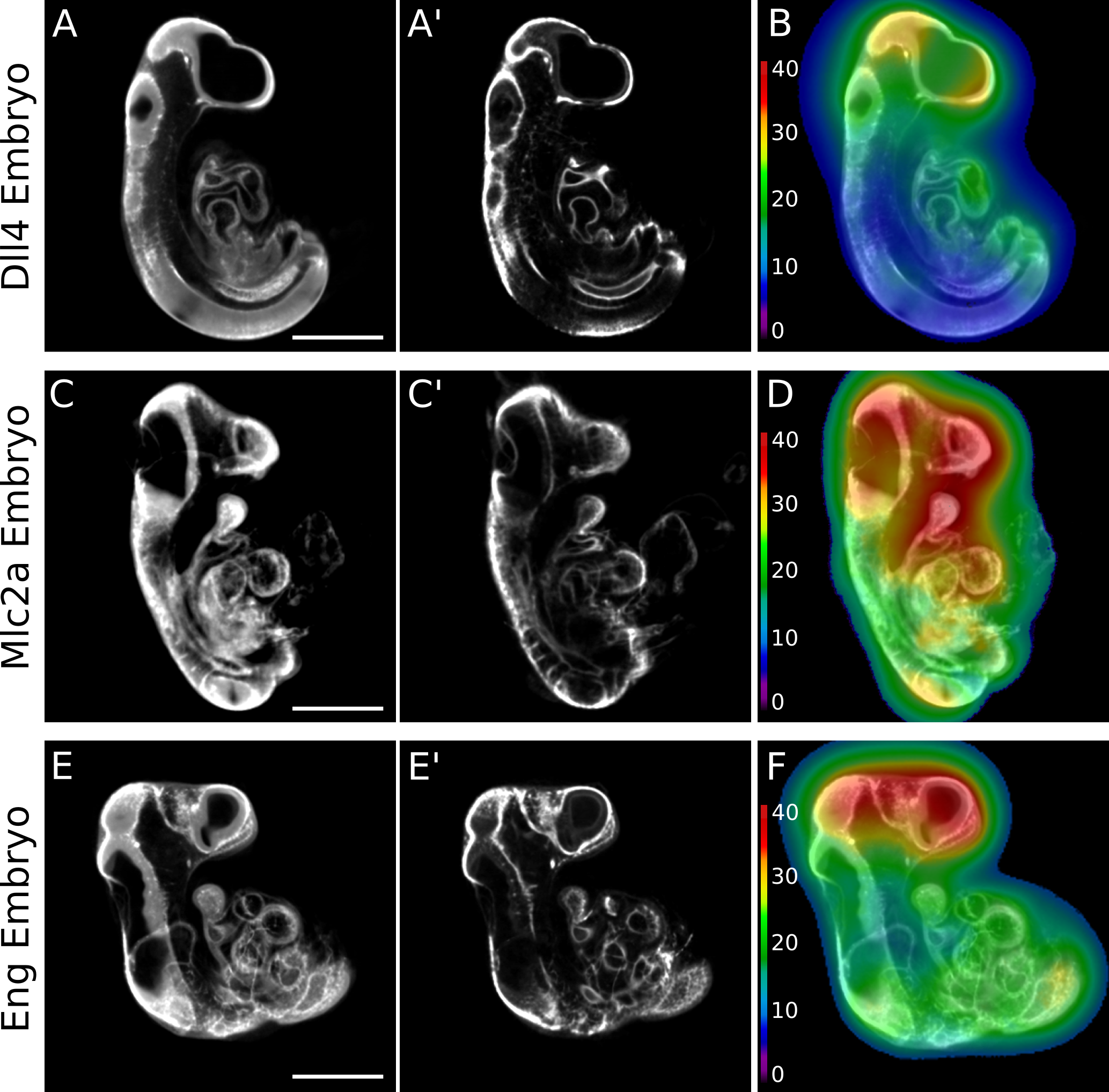

Supplement: S6 Fig — When a sagittal slice through the average autofluorescence image of the 25–28 somite Dll4 population average (A) or the average vasculature image (A′) is overlaid with the RMS displacement map generated in the registration pipeline, the movement of voxels required for proper alignment of the embryos can be visualized and quantified (B). The heat map scale bar (left side of B) indicates the voxel displacement in μm. The same is shown, respectively, for the 25–28 somite Mlc2a embryos (C, C′, and D), and the Eng embryos (E, E′, and F). Scale bar = 500 μm. (TIF) [file pone.0137175.s006.tif]
