# Supplementary material for: Cardiovascular Patterning as Determined by Hemodynamic Forces and Blood Vessel Genetics
Source: PLoS One. 2015 Sep 4;10(9):e0137175. doi: 10.1371/journal.pone.0137175 (PMC4560395; doi:10.1371/journal.pone.0137175)
Supplement: S1 Table — A table containing the information of all Dll4 embryos dissected during the course of this study. (PDF) [file pone.0137175.s007.pdf]

Supplemental Table 1 – Summary of all *Dll4* embryos dissected.

***Dll4* Mice**					
**LITTER**	**WT**	**HET**	**MUT**	**RESORP.**	**TOTAL**
1	3	6	3	2	14
2	5	0	0	4	9
3	6	4	0	1	11
4	1	5	1	3	10
5*	0	2	0	5	7
6	3	4	4	0	11
7	4	4	1	2	11
8	2	4	3	0	9
9	2	5	2	1	10
10	6	2	3	1	12
11	2	6	2	0	10
12	2	6	4	0	12
13	9	4	0	0	13
14	0	5	0	2	7
15	2	2	1	1	6
16	1	7	3	2	13
17*	1	0	1	0	2
18*	0	1	1	2	4
19*	0	1	1	0	2
20	4	4	1	0	9
21	5	3	2	0	10
22	2	6	1	1	10
**TOTALS**	60	81	34	27	202
**PERCENT**	30	40	17	13	

* - Indicates dissections in which only one uterine horn was implanted with embryos.
