# Supplementary material for: Cardiovascular Patterning as Determined by Hemodynamic Forces and Blood Vessel Genetics
Source: PLoS One. 2015 Sep 4;10(9):e0137175. doi: 10.1371/journal.pone.0137175 (PMC4560395; doi:10.1371/journal.pone.0137175)
Supplement: S2 Table — A table containing the information of all Mlc2a embryos dissected during the course of this study. (PDF) [file pone.0137175.s008.pdf]

Supplemental Table 2 – Summary of all *Mlc2a* embryos dissected.

***Mlc2a* Mice**					
**LITTER**	**WT**	**HET**	**MUT**	**RESORP.**	**TOTAL**
1	3	4	2	0	9
2	2	4	1	0	7
3	1	2	0	0	3
4	2	4	1	0	7
5§	5	8	2	0	15
6	2	5	3	0	10
7	0	6	0	0	6
8	1	1	3	0	5
9	2	3	4	0	9
10	3	3	2	0	8
11	5	4	1	0	10
12	8	2	2	0	12
13§	5	7	7	0	19
**TOTALS**	39	53	28	0	120
**PERCENT**	33	44	23	0	

§ - Indicates a combination of two litters of embryos.
