# Supplementary material for: Cardiovascular Patterning as Determined by Hemodynamic Forces and Blood Vessel Genetics
Source: PLoS One. 2015 Sep 4;10(9):e0137175. doi: 10.1371/journal.pone.0137175 (PMC4560395; doi:10.1371/journal.pone.0137175)
Supplement: S3 Table — A table containing the information of all Eng embryos dissected during the course of this study. (PDF) [file pone.0137175.s009.pdf]

Supplemental Table 3 – Summary of all Eng embryos dissected.

Eng Mice					
**LITTER**	**WT**	**HET**	**MUT**	**RESORP.**	**TOTAL**
1	0	4	4	0	8
2	1	5	2	0	8
3	2	6	0	0	8
4	4	4	0	0	8
5*	1	1	1	0	3
6*	1	2	0	0	3
7	4	7	2	0	13
8	1	5	2	0	8
9	3	5	0	1	9
10	1	2	1	0	4
11	0	1	2	2	5
12	2	2	1	1	6
13*	0	1	0	0	1
14	1	4	4	1	10
15	0	2	2	0	4
16	2	3	1	0	6
**TOTALS**	23	54	22	5	104
**PERCENT**	22	52	21	5	

* - Indicates dissections in which only one uterine horn was implanted with embryos.
